# Time and Place as Modifiers of Personal UV Exposure

**DOI:** 10.3390/ijerph15061112

**Published:** 2018-05-30

**Authors:** Brian L. Diffey

**Affiliations:** Dermatological Sciences, Institute of Cellular Medicine, University of Newcastle, Newcastle NE2 4HH, UK; brian.diffey@ncl.ac.uk

**Keywords:** UV index, time outdoors, holidays, outdoor leisure activities, solar altitude

## Abstract

It is a common belief that, if we want to limit our sun exposure during outdoor recreational activities and holidays in order to avoid sunburn or reduce our risk of skin cancer, we need to reach for the bottle of sunscreen or cover up with clothing. As important as these measures are, there is another way to enjoy our time outdoors and still benefit from the experience. In this article, we consider the impact of time, place, and behaviour outdoors on our exposure to solar ultraviolet (UV) radiation. Some of the simple actions we can take in controlling our UV exposure include being aware of the position of the sun in the sky, understanding how we can use the UV index to guide our outdoor exposure, and the importance of reducing our sun exposure around the middle of the day. Finally we review our preferred holiday activities and destinations, and the influence of outdoor leisure pursuits. By planning where and when we spend our leisure time in the sun, we can maximise our enjoyment whilst limiting our UV exposure.

## 1. Introduction

Most people believe that, if they want to limit their sun exposure during outdoor recreational activities and holidays in order to avoid sunburn or reduce their risk of skin cancer, they need to reach for the bottle of sunscreen or cover up with clothing. However, there is another way to enjoy our time outdoors and still benefit from the experience, and that is to consider how we might use time and place as ways to reduce the likelihood of overexposure. In other words, to plan where we spend our leisure time in the sun, and at what time of the day and year we can maximise our enjoyment whilst limiting our UV exposure.

Skin cancer prevention activities were first initiated in Australia from the 1960s, and later, coordinated primary prevention campaigns from the early 1980s (*Slip! Slop! Slap!*) were introduced by the State Cancer Councils with the objective of encouraging individuals to reduce their exposure to UV radiation. In 1988, the *Slip! Slop! Slap!* campaign was superseded by the more comprehensive *SunSmart* skin cancer prevention programme.

Beginning in 1993, the *Sun Know How* campaign was developed by the (now defunct) Health Education Authority in the UK with the aim of raising public awareness about the health consequences of overexposure to the sun. This campaign was replaced in 2003 by *SunSmart*, the UK’s national skin cancer prevention campaign, hosted until 2012 by Cancer Research UK with funding from the UK health departments. The *SunSmart* campaign incorporates research, public communication, working with professionals, and policy development to address the burden of skin cancer in the UK, and drew on the experiences of the Australian and other national and international health programmes.

In the United States, the Skin Cancer Foundation and the American Academy of Dermatology are major bodies active in sun awareness and skin cancer prevention, and many other countries can demonstrate similar activity by their health agencies.

This commentary addresses some of the temporal and spatial factors that influence personal UV exposure, refutes some common misconceptions, and illustrates simple ways of moderating behaviour to limit sun damage. However, we first need to understand the extent to which we are exposed to sunlight outdoors.

## 2. Time Spent Outdoors

How long we spend outdoors is a major contributor to our annual UV burden. The majority of people in developed countries mostly work indoors for 5 days per week, resulting in about 105 weekend days per year. In addition, if we include the number of paid vacation days (including public holidays), which is typically 28 days, this gives something like 133 days per year when there is the opportunity to be outdoors for extended periods. However, statutory paid leave varies considerably from zero days in the USA, 10 days in Japan, and up to around 40 days in, for example, Austria [1].

There are large variations between countries in the number of days that children spend at school each year. For example, in China children are at school for 260 days per year whereas in Bolivia the figure is 160 days [2]. A more typical value for many countries is around 190 days [2], leaving 175 days per year when there is the opportunity to spend many hours outdoors.

**Figure 1:**
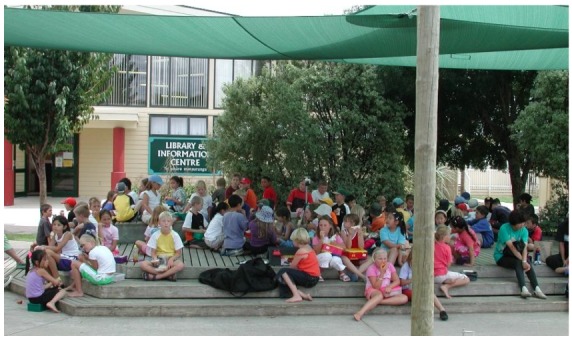
Schoolchildren in New Zealand having lunch outdoors. Note the shade provided by a canopy (courtesy of Christina Mackay, Victoria University of Wellington, New Zealand).

Unlike adults, children tend to have longer lunch breaks and often spend part of the school day outside engaging in activities such as physical education, as illustrated in Figure 1. This, coupled with more days away from the ‘workplace’ means that children have a much greater opportunity for sun exposure than adults.

Estimates of time per day spent outdoors generally come from one of two approaches: self-reported time using either personal diaries or recorded by time-stamped personal dosimeters, or recall studies asking people to estimate the time they spend outdoors during defined periods.

A large study of human activities in the United States using telephone interviews to collect 24-h retrospective diaries was reported in the National Human Activity Pattern Survey (NHAPS) [3]. This was a 2-year survey conducted on a virtually daily basis from late September 1992 through to September 1994 and involved a total of 9386 subjects. Interviews, which were completed in 63% of the households contacted, were conducted across 48 contiguous states and involved respondents of all ages.

**Figure 2:**
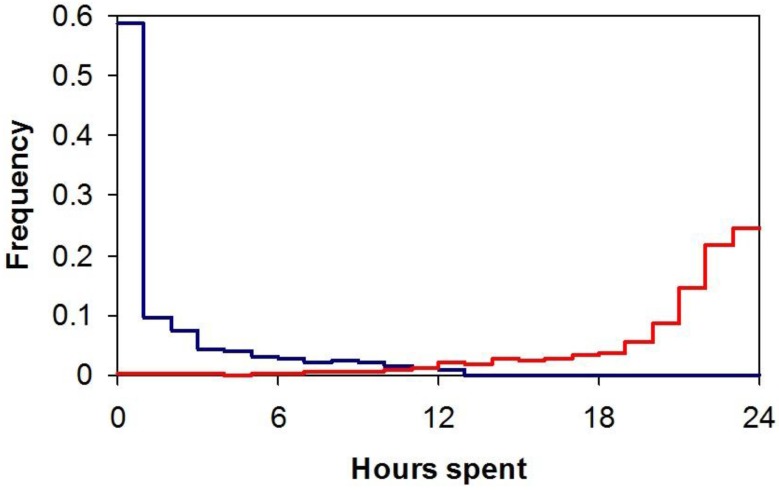
The distribution of times spent indoors (red) and outdoors (blue) by respondents in the National Human Activity Pattern Survey (NHAPS) study [3].

Respondents reported spending an average of 87% of their time in enclosed buildings, which included residence and workplace, and about 6% of their time in enclosed vehicles, with only 7% of time outdoors. The percentage of time spent indoors, outdoors, and in vehicles was, on average, fairly invariant across people in different parts of the USA [3]. The frequency distribution of times spent both indoors and outdoors is shown in Figure 2.

Note that time spent outdoors is highly positively skewed compared with a negative skew for time spent indoors. Whilst 99.9% of respondents spent some time indoors during the 24-h period for which they kept a diary, only 59% of people spent one minute or more outdoors and so the large amplitude between 0 and 1 h for the outdoor plot includes those subjects who never went outdoors during the 24-h diary period.

Just how heterogeneous time outdoors can be was demonstrated in an overview analysis [4] of the median times per day spent outdoors by adults from the USA and several European countries during weekdays and weekends, as recorded in a number of different studies either by diaries, dosimeters, or by recall.

There will be many sources of heterogeneity between these studies, which include differences in geographical location, duration, time period during the day, and months of the year when sampling took place. There will also be differences in the attributes of the cohorts in each study such as age, gender, physical activity level, health, and the employment status of individuals. Additionally, weather conditions, such as daily maximum temperature and precipitation, will also contribute to the variance.

Notwithstanding the heterogeneity that exists between the various studies, the pooled estimates of median times per day outdoors were around 1.0 h and 1.5 h for weekday and weekend exposure, respectively [4]. The pattern of time outdoors for holiday exposure more closely followed a normal distribution with a mean time per day outdoors of about 5 h. Although these summary values should be regarded cautiously, they may still provide the best estimate of exemplary values for the population than using the data obtained in any single study (Stein’s paradox [5]).

## 3. Behavioural Influences on Exposure to Solar UV Radiation

Factors that influence our exposure to the sun include:

- Predispositions—knowledge, attitudes, beliefs, and aspirations
- Social norms—our desire to conform with others whose opinions we value
- Physical environment—ambient UV, weather, and the availability of shade
- Demands of the activity we intend to engage in—for example, it is easier to adopt protective measures sitting under a tree reading a book than it is swimming in the sea.

In addition, many of these factors will be contingent on our genetic susceptibility to sun damage (sun-reactive skin type [6]) and our phenotype characteristics (e.g., hair colour, freckles, etc.).

**Figure 3:**
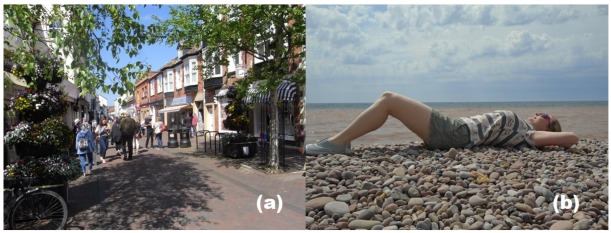
(**a**) Adventitious sun exposure and (**b**) elective sun exposure.

Broadly speaking, we can divide our sun exposure into *adventitious* exposure, typified by the unavoidable exposure associated with activities such as shopping and travelling to work where exposed sites are normally limited to the face, neck, and hands (Figure 3a), and *elective* exposure, when we deliberately go to seek the sun for recreational purposes, usually during summer weekends and holidays. During our elective exposure we often expose our arms, legs, and sometimes our trunk or even whole body (Figure 3b).

Both measurement and modelling show that for many people, especially those who live at temperate latitudes, about three-quarters or more of their annual solar UV burden results from holiday and recreational exposure during the summer (May to August in the northern hemisphere) [6]. This is not surprising given that for many people holiday exposure occurs in sunny locations when they are outdoors for several hours each day around noon often with little or no shade.

## 4. Informing the Public on UV Levels

Meteorological services in many countries provide an important role in providing the public with information on the expected UV Index (UVI) [7] for the current and forthcoming days. In estimating the UVI, forecasters take into account the solar altitude, ozone amounts in the stratosphere, and the expected cloud cover.

When the UVI is 2 or less, there is little or no risk of sunburn apart from in people who are pathologically photosensitive. At UV Indices of 3–4 on the skin, exposure times need to be around one hour on unacclimatised white skin before there is a need for photoprotection such as sunscreen, clothing, or shade [6]. Exposure times before protective measures are needed will shorten as the UVI increases towards 9–10, and from indices of 11+, delayed erythema may result from as little as 10 min of unprotected exposure.

Traditional media, such as magazines, newspaper, radio, and television, play an important role in disseminating information related to sun protection, especially the role of sunscreen, and it is through these channels that most people learn about UV effects on the skin and photoprotection.

More recently, the internet and social media have supplemented the messages from traditional media with the availability of websites and apps related to, for example, sun safety, the UVI and when to re-apply sunscreen. Some of these apps use satellite data to determine an individual’s location, and use these data to estimate the UVI followed by giving personal sun exposure advice based on solar UV intensity and the individual’s skin type, determined in response to answers to questions requested by the app.

**Figure 4:**
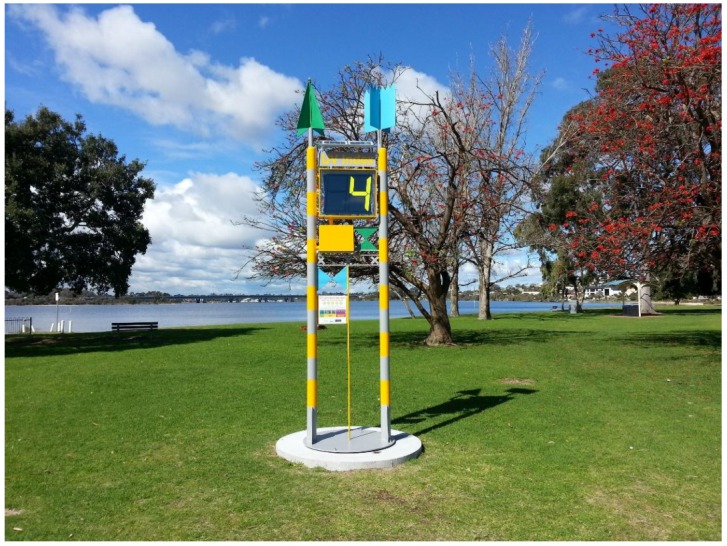
A UV meter displaying the live UV Index at Deep Water Point Reserve in Western Australia (Image courtesy of Cancer Council Western Australia).

Other innovations include placing UV meters in public areas (Figure 4). Initiatives such as this are designed to provide a live reading of UV Index for that location helping people ensure they are taking the correct measures to protect themselves from the sun.

In addition to indicating when sun protection is needed, the UV meters are also an educational tool for people to learn about the UV Index and what it means. UV meters such as this do require individuals to have some self-awareness of their own skin type and propensity to burn, and to use this self-knowledge in tandem with the UVI data.

## 5. Simple Actions to Controlling Our UV Exposure

Whilst external agencies can educate and guide our behaviour in the sun, the most significant contribution to an individual’s solar UV burden is personal choice.

### 5.1. Time, Place, and Position of Sun in the Sky

We are most at risk of overexposure under a cloudless sky and on days such as these, it is the position of the sun in the sky that is the major determinant of ambient UV levels. The sun rises in the east, reaches maximum height at solar noon in the south (in the northern hemisphere), and sets in the west. (In the southern hemisphere, maximum solar elevation is in the north). The local time when solar noon occurs depends on longitude and whether daylight saving time is in force. For example, on midsummer day (21 June) in Madrid (latitude 40.4° N, longitude 3.7° W) solar noon occurs at a local time of 14:16 p.m., whereas in Berlin (latitude 52.5° N, longitude 13.4° E), which is on the same time zone as Madrid, solar noon on this date occurs at a local time of 13:08 p.m.

**Figure 5:**
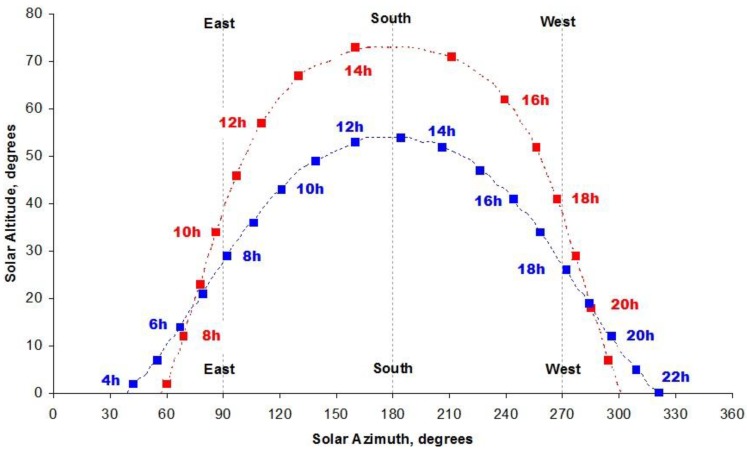
The sun path in Stockholm (blue) and Madrid (red) on 21 June. Time is given in local (daylight saving) time, which is 2 h ahead of GMT, and the solar azimuth angle is expressed relative to true north.

The sun path, which refers to the seasonal and hourly positional changes of the sun as the Earth rotates and orbits around the Sun, can be determined at any latitude and any time of the year from astronomical geometry, and Figure 5 shows an example for two European cities on the same time zone on 21 June: Stockholm (latitude 59.3° N, longitude 18.0° E) and Madrid (latitude 40.4° N, longitude 3.7° W).

Note that the sun reaches a greater solar altitude in Madrid but that the daylength of about 15 h is shorter compared with 18 h in Stockholm. On-line software is available to calculate and plot the sun path for chosen dates and locations (http://sunposition.info/sunposition/index.php?sunposition).

### 5.2. Shadow Rule and UV Index

A basic technique that can be used as a rough guide to the need for sun protection is to apply the so-called *Shadow Rule* [8]. Put simply, if your shadow is longer than your height (i.e., solar altitude < 45° in Figure 5), which occurs typically in the early morning and late afternoon, your UV exposure is likely to be low and sun protection is generally not required. However, if your shadow is shorter than you are (i.e., solar altitude > 45° in Figure 5), typically around the middle of the day, the levels of UV radiation are sufficiently high that you would be advised to seek shade and protect your skin and eyes. Of course, the shadow rule only works if the sun is not covered by cloud, and principally applies to sun exposure at or near sea level.

**Figure 6:**
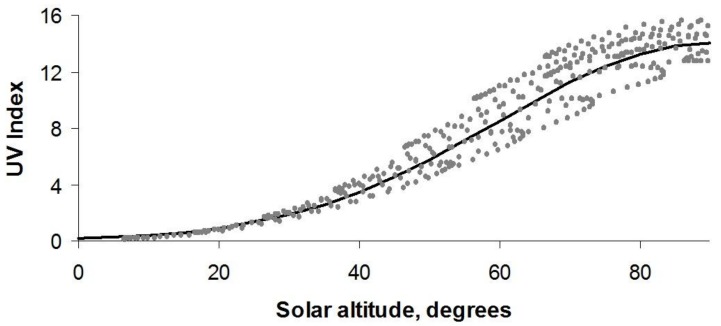
The variation of UV Index with solar altitude.

The rationale for this rule is that solar altitude is the main determinant of solar UV intensity, which is generally expressed by the UVI. The maximum UVI, which occurs at solar noon, determined for different cloudless days throughout the year and at latitudes ranging from 0° N to 60° N [9] is plotted against the calculated solar altitude for the corresponding day and latitude in Figure 6.

We see that UVI increases as the sun rises higher in the sky. There is variation in UVI around a given solar altitude due to temporal and geographic variations in ozone thickness. However, the relationship can be roughly expressed as

UVI = 14 × exp{−[(90 − *A*)^2^/1800]}

where *A* is the solar altitude in degrees. This expression is shown by the solid curve in Figure 6. From this expression we estimate that when your shadow length equals your height—that is, a solar altitude of 45°—the UVI is typically around 4.5.

Interpretation of the UVI is dependent on an individual’s propensity to sunburn, as illustrated in Table 1.

When the risk is low, little needs to be done in the way of sun protection but as the risk rises then increasing vigilance and protection measures are needed. When shadow length is one-half or less of a person’s height, UV levels are very high and considerable care needs to be taken. It should be stressed that the solar altitude and corresponding shadow length ranges given in Table 1 are simply a guide since, in addition to solar altitude, factors such as ozone thickness and altitude, with possible reflection from snow, will have some bearing on UV exposure of body surfaces.

### 5.3. Sun Avoidance around the Middle of the Day

Solar UV intensity is highest in the 3-h period around local noon when 40–50% of a summer’s day UV is received over the latitude range 10° N to 50° N. At more northerly latitudes, the long daylength in the summer means that only 25% of diurnal erythemal UV is received in this 3-h period at 60° N.

The classic example of avoiding the sun around the middle of the day is the *siesta*, which is a short nap taken in shade, often after the midday meal. The siesta is a common tradition in warm countries, especially throughout the Mediterranean and Southern Europe, where museums, churches, and shops normally close for 1 to 3 h from midday to early afternoon so that workers can go home for a long lunch and perhaps a sleep during the day’s hottest hours.

The health benefit of a siesta habit is not just limited to a reduced UV exposure during the hours of highest insolation, but also a short sleep after lunch is said to reduce stress, help cardiovascular function, and improve alertness and memory [10].

**Figure 7:**
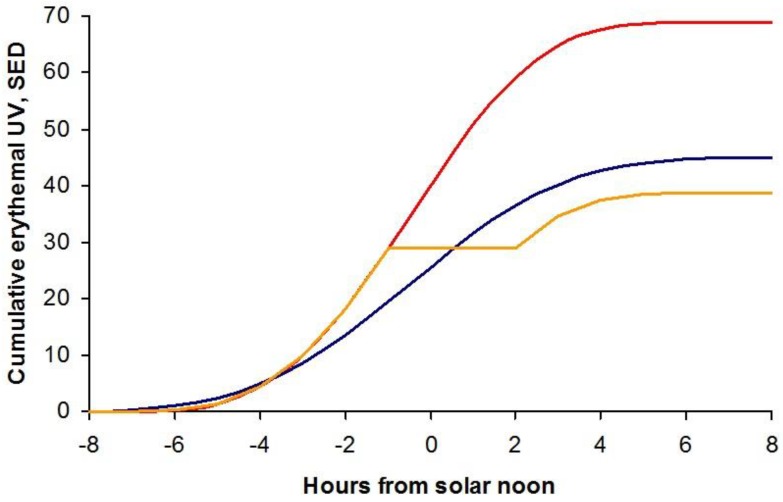
Cumulative ambient erythemal UV throughout a cloudless summer day in Tenerife (red curve) and Edinburgh (blue curve). Avoiding the sun for 3 h around the middle of the day in Tenerife is shown by the (orange curve).

So clearly avoiding direct sunlight around the middle of the day can be an effective first step in limiting personal exposure. This is illustrated in Figure 7, which shows the cumulative ambient UV throughout a cloudless summer day in Tenerife (28° N) and Edinburgh (56° N).

By the end of the day the cumulative ambient UV in Tenerife is 69 SED compared with 45 SED in Edinburgh. (The Standard Erythema Dose (SED) is a measure of erythemal UV radiation [11]; 1 SED is equivalent to an erythemally-weighted dose of 100 J m^−2^.) But if a leisurely 3-h lunch break is taken when on holiday in Tenerife, the cumulative UV at the end of the day, shown by the orange curve in Figure 7, is less than received during a full day in Edinburgh.

## 6. Holiday Sun Exposure

If the goal is to reduce our annual UV burden without curtailing holiday or outdoor leisure activities, we need to consider when and where to take vacations, what time of day to limit sun exposure, and how to spend our time outdoors. Over-concern about our exposure during the 6-month period October through to March at temperate latitudes in the northern hemisphere will have little impact on our overall annual exposure. Of course, if a winter-sun holiday is taken either in a location close to sea level at latitudes between about 30° N to 30° S, or in the opposite hemisphere, then sun protection measures may be necessary.

For this reason, the discussion that follows deals solely with summertime activities and although winter vacations, especially to snowfields, are popular, they generally contribute little to annual UV exposure due to a combination of low ambient UV levels and almost full body coverage by clothing. The critical organ for exposure at high altitudes in snowfields is the eye and appropriate ocular protection is a sensible precaution under these conditions, as is sun protection for the face and any other exposed body parts, particularly towards the end of the winter season when the sun becomes stronger and the environment remains highly reflective.

In a review such as this, where we are considering temporal and spatial modifiers of personal UV exposure, it is not possible to give an exhaustive appraisal of holiday habits of several populations. Instead, we use as an example the holiday patterns adopted by British people, and presume similar behaviour by people from other countries in temperate regions.

In today’s world lifestyles are changing in many ways, none more so than the opportunity for overseas travel. The total number of visits abroad by UK residents in 2016 was almost 71 million and of these 45 million were for holidays [12].

**Figure 8:**
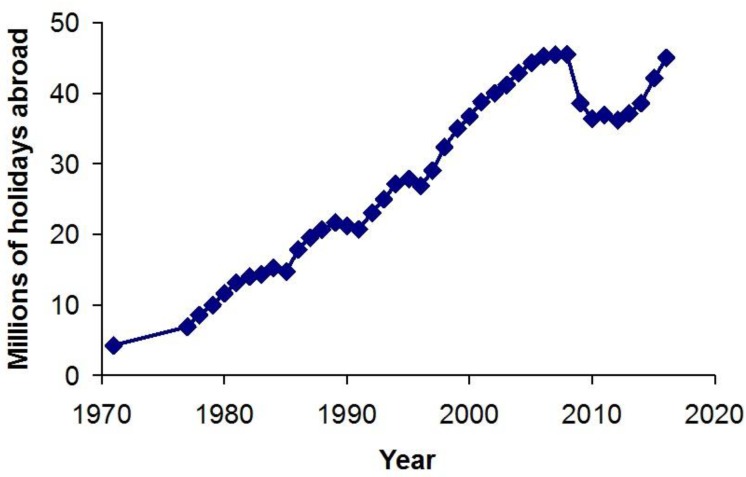
Holidays abroad taken by UK residents in the period 1971–2016 [12].

Nearly two thirds of visits abroad by UK residents are for vacations, with a 10-fold increase in the number of overseas holidays taken by British residents in the period 1971–2016. The number of holidays overseas peaked at just over 45 million in 2008, dropped by 16% the following year possibly as a consequence of the economic downturn, but are now on the rise again (Figure 8).

Holiday travel by British people, and probably by residents of other northern European countries, are overwhelmingly to more southerly destinations where UV levels are typically high. For example, Spain continued to be the top destination for UK residents visiting abroad in 2016, accounting for 13 million visits or almost 30% of the total number of foreign holidays.

Almost 90% of Britons reported [13] taking a holiday either at home or abroad in the 12 months up to August 2017; the largest percentage since 2011. The average number of holidays in 2017 was 3.8 per person, an increase of 0.4 on 2016, with the increase being driven by both domestic and foreign holidays.

British people took an average of 1.7 overseas holidays in the 12-month period to August 2017 with 57% of people holidaying abroad. The average person took 2.1 domestic holidays, with 72% of British people having taken a UK break in the last 12 months [13].

As we move through life, our desire/ability to take a holiday changes, with families typically taking more holidays than other groups. Whilst the number of overseas holidays remains relatively consistent across the life stages, families, particularly those with older children, are the most likely to take a domestic holiday, going on an average of 2.5 trips per person [13].

The ten most popular types of holiday taken by British people in the 12-month period to August 2017 are given in Table 2 [13].

City breaks overtook beach holidays to become the nation’s favourite holiday type in 2014, a position they have held since then. Beach holidays, where the potential for overexposure leading to sunburn is high, remain the second most popular type of holiday [13].

Clearly changing patterns of holidaymaking are an important factor that has increased the overall UV burden received by many populations from more northerly latitudes in recent decades.

A summer holiday to a sunny destination can contribute a substantial fraction of our annual UV burden and one way to reduce this is to go on holiday (in the northern hemisphere) between October and March. As an example, the maximum UVI in Tenerife in June/July is 12 and daytime temperatures often reach 30 °C. In January, the weather is still pleasantly warm with average daytime temperatures between 16–21 °C but the maximum UVI is substantially lower at around 5.

Other options include winter ski resorts, choosing summer holiday destinations in temperate rather than tropical latitudes, and/or opting for sightseeing holidays in preference to beach holidays whilst taking advantage of the shade provided by churches, galleries, museums, and other buildings.

## 7. Outdoor Leisure Pursuits

Not only can holidays to sunny destinations result in substantial UV exposure, but overexposure can also be a consequence of outdoor recreational pursuits, especially those requiring an extended period outside such as a round of golf.

Choosing to participate in outdoor leisure activities is affected by a number of demographic and socio-economic factors that include age, sex, ethnicity, and health, as well as economic and family circumstances. Less obvious influences include peer group participation, participation as a child, and where people live relative to available facilities.

A detailed overview of the engagement of British adults in sport [14] found that men and women are equally likely to walk for health and recreational purposes, although men are more likely to cycle or to take part in outdoor sports. Participation in sport is also higher among younger people and those in higher income bands. The latter observation may be a contributory factor to the greater incidence of melanoma in those of higher socio-economic status.

In the four weeks prior to interview for the participation in sport survey [14], 68.0% of people walked and 10.3% cycled for health and recreation. The frequency with which people participate varies by activity. For example, 25% of people walk four or more times a week but only 11% cycle this often.

**Figure 9:**
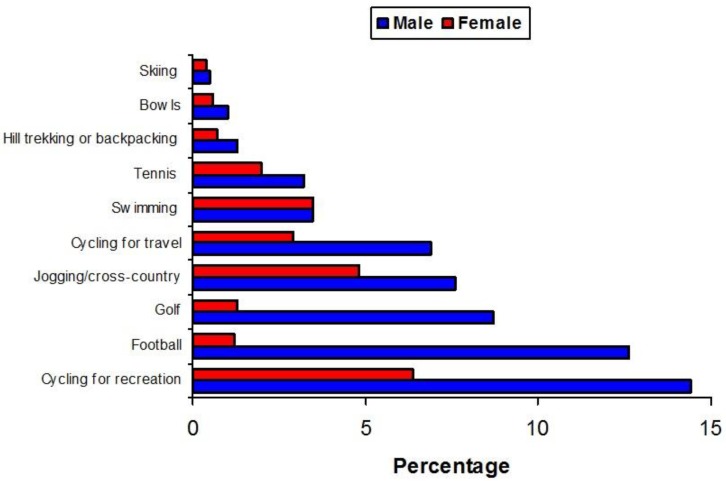
The percentage of British people who engage in various outdoor recreation pursuits [14].

Whilst men are more likely than women to engage in outdoor sports (Figure 9), indoor activities such as ice skating, keep fit, aerobics, and yoga are more likely to be taken up by women.

Similar findings to this British survey are seen in the American population [15]. In 2016, 144.4 million Americans, or nearly half of the US population, participated in an outdoor activity at least once, with the most popular activities being running/jogging (18% of Americans), angling (16%), cycling (15%), and hiking (14%) [15].

Walking for fitness was, by far, the most popular activity and undertaken by 45% of all outdoor participants. There has been little in the way of a trend in outdoor activity with between 48% and 50% of all Americans aged 6 years and upwards engaging in outdoor activity in the period 2007–2016.

**Figure 10:**
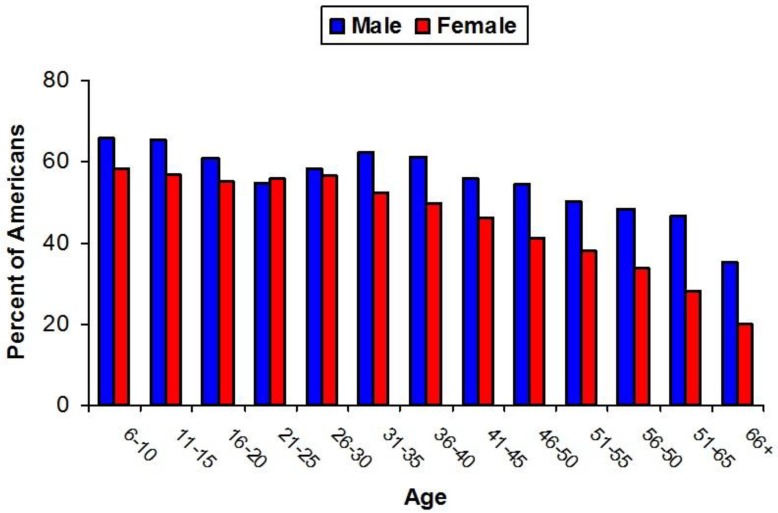
The percentage of American people by age and gender who engage in outdoor recreation pursuits [15].

Participation in outdoor recreation varies as individuals age, with gender also playing a role in determining behaviours and participation trends (Figure 10).

Outdoor activities are popular among children, but participation rates drop for both males and females from the late teenage years to the early 20s and then climb back up slightly for females in their late 20s and males in their 30s before gradually declining throughout life.

An effective strategy to minimise UV exposure when engaging in sporting and other activities outdoors is to schedule the activity, if possible, during early morning or late afternoon/evening. It is preferable to commence the activity in the afternoon rather than the morning since, if the event runs on longer than expected, the UV exposure will be decreasing with time in the afternoon, unlike in the morning when the UV Index is increasing up until solar noon.

Another consideration in planning an outdoor activity is, if possible, to ensure that there is adequate existing shade and/or providing temporary shade structures, for example, a sun umbrella whilst angling on a river bank or a canopy over an outdoor swimming pool.

### 7.1. The Influence of Ambient Temperature on Health

An important factor to consider when scheduling outdoor sporting activities is the impact that high temperatures, sometimes coupled with high humidity, can have on the performance and health of the participants. For example, the 2018 Australian Open Tennis Tournament held in Melbourne during the last fortnight of January 2018, resulted in players and fans enduring blistering heat that sent temperatures soaring to around 40 °C on some days.

**Figure 11:**
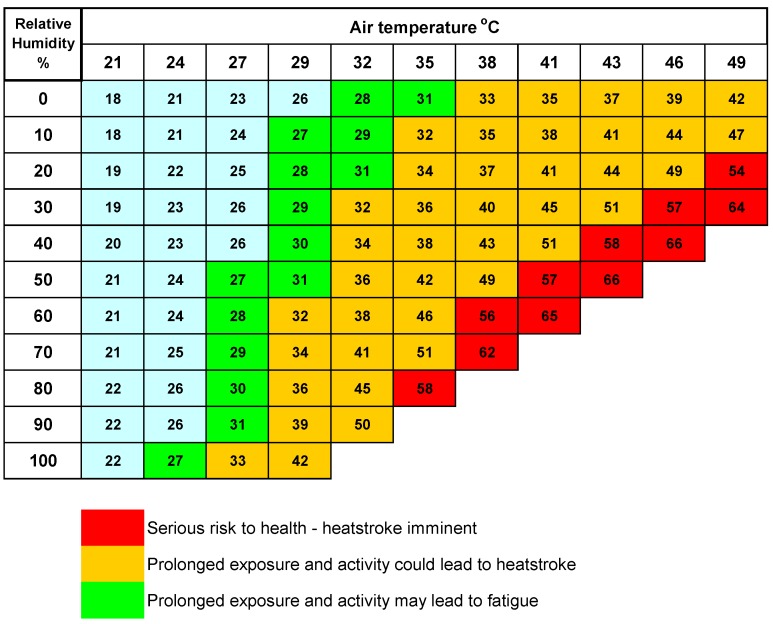
Apparent temperature (heat index) in degrees Celsius according to air temperature and relative humidity [16].

Many major sporting events operate an extreme heat policy. In this case of the Australian Tennis Open, the policy goes into effect once the ambient temperature exceeds 40 °C and the Wet Bulb Globe Temperature (WBGT) exceeds 32.5 °C. The WBGT is a measure of the heat stress in direct sunlight, which takes into account temperature, humidity, wind speed, solar altitude, and cloud cover. This differs from the heat index (see Figure 11), which indicates how hot it feels and is expressed as a function of ambient air temperature in the shade and the relative humidity.

When the temperature is high but the relative humidity is low, the heat index is less than the actual temperature. This is because cooling by evaporation of sweat is very efficient in these situations. However, when the relative humidity is high, evaporation is prevented and so it seems hotter than it really is. A combination of high temperature and high humidity leads to extreme heat indices and in these situations, physical activity outside can lead to heatstroke or even death.

### 7.2. Ambient Temperature and Sunburn

Ambient temperature influences the magnitude of individual solar UV exposure and the consequent risk of sunburn. In a behavioural study in Australia [17], it was observed that the likelihood of sunburn approximately doubled when the ambient temperature was in the range 19–27 °C, compared with either lower or higher temperatures. The reason for this is presumably because warm temperatures encourage people to spend more time in direct sunlight with the increased risk of sunburn. Yet at temperatures in excess of 27 °C, it was observed [17] that the likelihood of sunburn fell as people became more aware of the need for extra precautions or simply sought shade for comfort reasons.

On days when high temperatures are expected, weather forecasters frequently warn about the dangers of UV and high UV indices. It is not surprising, therefore, that it is a common belief that high ambient air temperature is a major risk factor for burning. Although the UV index is generally higher on cloudless, hot days compared with cloudy, cool days, reliance should not be placed on ambient temperature alone as a guide to the need for sun protection.

**Figure 12:**
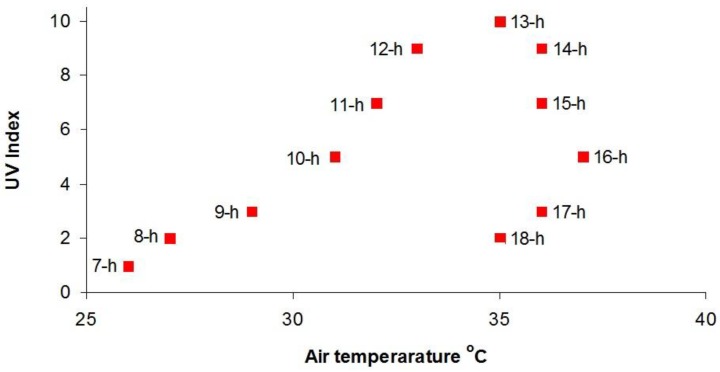
The air temperature and UV index in hourly intervals at Melbourne, Australia on 28 January 2018.

This is illustrated in Figure 12, which shows the UVI and air temperature, obtained from an online weather service [18], in hourly intervals at Melbourne, Australia (latitude 37.8° S, longitude 145.0° E, 31 m asl) on 28 January 2018, which was a sunny day where the solar altitude reached 71 degrees and solar noon occurred at the local time of 13:32 p.m.

We see that in the morning both the air temperature and UVI increased steadily but in the afternoon the temperature continued to rise until mid-afternoon, whereas the UVI fell steadily. By 18-h, the UVI was only 2 and presented little risk of sunburn yet the air temperature was still in the mid-30s.

It is not uncommon to see people apply sunscreen in the late afternoon/early evening when it may still be very hot. However, in mid-summer the UVI 4 h after solar noon is between 2 and 3 and one hour later it is between 1 and 2 at latitudes between the Arctic and Antarctic Circles. At these UV indices, there is little risk of sunburn.

Consequently it is unwise to judge the erythemal power of the Sun solely on ambient temperature. This is true not only when it is very hot but also when it is cloudy, or there is a cool breeze, as the risk of overexposure may be increased because the warning sensation of heat is diminished.

Average seasonal temperatures are often, but not always, positively associated with ambient UV levels and so, by organising events to minimise heat load on participants, both the sportsmen and viewing public are likely to benefit from lower UV exposure.

One example of re-scheduling sporting events is the 2022 FIFA World Cup due to be held in Qatar. Traditionally this event is held in June and July but because of high summer temperatures that typically average around 42 °C, the event will be played in November and December when typical average temperatures are about 24 °C. The maximum UVI in Qatar in the summer is typically 11 or higher but, at the time the competition is scheduled for, it will be around 6.

### 7.3. Sun Exposure at the Coast

Spending time at the coast is popular both on holiday and for recreational activity, especially swimming. Many people believe that they sunburn easily at the coast because of high reflectance of sunlight from the sea, which is perhaps not surprising given the conventional wisdom that water, along with snow and sand, has a high reflectance for solar UV radiation and that articles in popular magazines commonly use phrases such as “Water is a highly reflective surface...” [19]. Figures quoted for the reflection of UV from water vary from about 5% [20] up to around 30% [21].

The reflected solar UV radiation from the ocean surface comes from two processes. Some of the direct and diffuse solar radiation incident on the surface enters the water there to be scattered one or more times until part of that radiation is re-emitted from the water and part is absorbed or transmitted to deeper depths. The remainder of the incident radiation is reflected upward by the sea surface due to the difference in the refractive indices of air and water. The relative contributions of these two effects depend on the constituents of the ocean in terms of phytoplankton, dissolved substances, mineral particles, bubbles, etc., on the roughness of the sea surface (the wave state, usually characterised by the wind speed), and on the angular distribution of the incident direct and diffuse UV radiation. However, it is UV reflected at the sea surface that is the dominant contribution to upwelling radiation and typically comprises 80% or more of the total reflected solar UV from the sea.

Irrespective of the height of the sun in the sky and the direction a subject is facing with respect to the sun, the contribution of UV radiation resulting from surface reflectance and emerging from the sea (backscattered), is never more than equivalent to a UVI of 0.7 [22]. For a sunbather, who is lying supine on a beach or the deck of a boat, there is no contribution from UV radiation reflected from the ocean to their erythemal exposure. It seems, therefore, that the reason people get sunburnt at the seaside has more to do with the absence of shade due to structures such as buildings or trees, which often block much of the sky in urban areas, than with reflectance by the water surface or even beach sand, which typically has an erythemal reflectance of around 15% [20].

**Figure 13:**
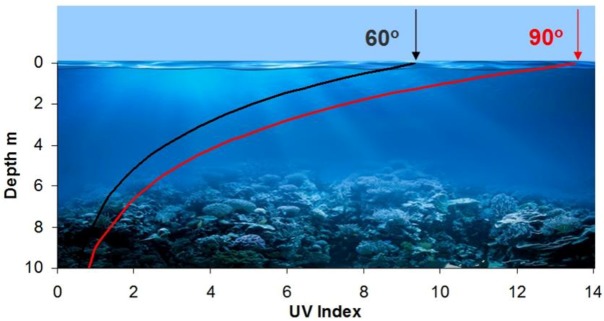
UV indices on a horizontal surface, e.g., the back of a swimmer, at different depths in a typical ocean for solar altitudes of 90° (red curve) and 60° (black curve) corresponding to above-surface UV indices of 13.6 and 9.5, respectively, under clear sky conditions (modified from reference [23]).

Sunburn is a real possibility when swimming in the ocean as UV will penetrate several meters into seawater, the degree of attenuation depending on the concentration of chlorophyll and other absorbers. This is illustrated in Figure 13, which shows how the UVI changes with depth in open oceans with a typical chlorophyll concentration of 1 mg m^−3^ for solar altitudes of 90 and 60 degrees.

For very clear waters where the chlorophyll concentration is typically around 0.05 mg m^−3^, such as a tropical coral reef, alpine lake, or swimming pool, and the sun is high in the sky, the UVI within the water can be greater than 10 at depths down to two meters, and greater than 6 down to 5 m. Thus, snorkelling in clear water at a depth of 5 m for 30 min or more may result in sunburn in susceptible individuals.

## 8. Conclusions

The cornerstones of radiation protection are administrative control measures, engineering control measures, and personal protection. By administrative control measures in the context of sun protection, we mean the information and knowledge that enables a personal, informed choice of behaviour outdoors; engineering control is directed at the provision of shade, as exemplified by the canopy shown in Figure 1; and personal protection measures are principally clothing and the application of topical sunscreen.

This commentary has limited itself to a consideration of so-called administrative control measures. In other words, the information provided by public health agencies, in addition to print, broadcasting, and social media, that is aimed at providing us with the data necessary to make an informed choice about when and where to spend time in the sun. An appreciation and understanding of the changing levels of ambient solar UV radiation with geography, season, and time can enable us to maximise our enjoyment of the sun whilst limiting cutaneous damage.

## Figures and Tables

**Table 1:** The risk of sunburn and skin damage as a function of UV index for different skin types, together with the approximate range of solar altitudes and shadow lengths corresponding to the UV indices.

UV Index	Solar Altitude	Shadow Length Relative to Height	Sun-Reactive Skin Type		
			I/II	III/IV	V/VI
0	<20°	>2.7	Very low	Very Low	Very Low
1–2	20°–35°	2.7–1.4	Low	Low	Low
3–4	35°–45°	1.4–1.0	Medium	Low	Low
5–6	45°–55°	1.0–0.7	High	Medium	Low
7–10	55°–65°	0.7–0.5	Very high	High	Medium
11+	>65°	<0.5	Extreme	Very high	High

**Table 2:** The ten most popular types of holiday taken by British people in the 12-month period up to August 2017 [13].

Holiday Type	%
City break	53
Beach holiday	41
Countryside break	25
All-inclusive holiday	17
Trip to an event	9
Trip to lakes and mountains	9
Renting a private home or house swap	9
Activity holiday e.g., walking, cycling	7
Cruise	7
Coach holiday	6
